# Comparative Prognostic Value of AFP, PIVKA-II, and GPC-3 in Hepatocellular Carcinoma: A Real-World Multicenter Cohort Study

**DOI:** 10.3390/biomedicines14102146

**Published:** 2026-09-23

**Authors:** Francesco Damone, Gian Paolo Caviglia, Piero Colombatto, Filippo Oliveri, Marta Guariglia, Gabriele Ricco, Veronica Romagnoli, Daniela Cavallone, Emanuela Rolle, Patrizia Carucci, Francesco Faita, Ferruccio Bonino, Silvia Gaia, Maurizia Rossana Brunetto

**Affiliations:** 1 Hepatology Unit and Laboratory of Molecular Genetics and Pathology of Hepatitis Viruses, Reference Center of the Tuscany Region for Chronic Liver Disease and Cancer, University Hospital of Pisa, 56124 Pisa, Italy; f.damone@studenti.unipi.it (F.D.); p.colombatto@ao-pisa.toscana.it (P.C.); f.oliveri@ao-pisa.toscana.it (F.O.); riccogabriele@gmail.com (G.R.); v.romagnoli@ao-pisa.toscana.it (V.R.); danielacavallone@hotmail.com (D.C.); 2 Department of Medical Sciences, University of Turin, 10126 Turin, Italy; gianpaolo.caviglia@unito.it (G.P.C.); marta.guariglia@unito.it (M.G.); 3 Gastroenterology Unit, Città della Salute e della Scienza—Molinette Hospital, 10126 Turin, Italy; emanurolle@inwind.it (E.R.); pcarucci@cittadellasalute.to.it (P.C.); silvia.gaia74@gmail.com (S.G.); 4 Institute of Clinical Physiology, Italian National Research Council (IFC-CNR), Via Moruzzi, 1 56124 Pisa, Italy; francesco.faita@cnr.it (F.F.); ferruccio.bonino@unipi.it (F.B.); 5 Department of Clinical and Experimental Medicine, University of Pisa, 56126 Pisa, Italy

**Keywords:** Hepatocellular carcinoma, serum tumor markers, alpha-fetoprotein, protein induced by vitamin-K absence-II, glypican-3, overall survival

## Abstract

**Background/Objectives**: The current cornerstone for prognostic hepatocellular carcinoma (HCC) stratification is the Barcelona Clinic Liver Cancer (BCLC) score, which integrates tumor and liver function staging but lacks tumor biology parameters. Serum tumor biomarkers, such as alpha-fetoprotein (AFP), protein induced by vitamin K absence-II (PIVKA-II), and glypican-3 (GPC-3), may improve prognostic assessment. We evaluated their prognostic value in this multicenter retrospective study. **Methods**: Serum AFP, PIVKA-II, and GPC-3 levels were measured (as continuous and categorical variables: >20 ng/mL, >200 mAU/mL, and >150 pg/mL, respectively) at baseline in 605 consecutive patients newly diagnosed with HCC at two tertiary Italian centers (2010–2024). The correlation between tumor stage, liver function, tumor marker levels, and overall survival (OS) was analyzed using Kaplan–Meier and Cox proportional hazards models. **Results**: Tumor marker levels increased stepwise across BCLC stages. Median OS was 29.0 months, declining from 47.9 months (BCLC-0) to 10.7 months (BCLC-C; *p* < 0.001). In univariate analysis, shorter OS was significantly (*p* < 0.001) associated with elevated serum levels of all tumor markers, higher ALBI grade, presence of ascites, and portal hypertension. On multivariate analysis, BCLC stage, ascites (hazard ratio [HR] 2.19, *p* < 0.001), ALBI grade 2–3 (HR 1.36, *p* = 0.009), and PIVKA-II > 200 mAU/mL (HR 1.56, *p* < 0.001) were independently associated with OS, whereas GPC-3 showed a borderline association (HR 1.27, *p* = 0.058), but not AFP (HR 0.88; *p* = 0.306). **Conclusions**: In this multicenter retrospective cohort study, PIVKA-II was the only serum biomarker independently associated with OS after adjusting for BCLC stage and liver function. These findings support further prospective evaluation of whether PIVKA-II provides clinically meaningful incremental prognostic information beyond established BCLC-based assessment.

## 1. Introduction

Hepatocellular carcinoma (HCC) accounts for approximately 90% of primary liver cancers, represents a major global health problem, and is the third leading cause of cancer-related mortality worldwide [1,2]. Despite major advances in surveillance, diagnosis, and treatment, prognosis remains highly heterogeneous, reflecting the complex interplay between tumor burden, hepatic reserve, and intrinsic tumor biology [3,4].

The Barcelona Clinic Liver Cancer (BCLC) staging system integrates tumor burden, liver function, and performance status and represents the cornerstone for therapeutic decision-making and prognostic stratification [5]. However, BCLC staging alone does not fully capture tumor biological aggressiveness, and additional parameters reflecting molecular and functional heterogeneity are needed to refine the risk assessment and guide personalized management [6]. In other malignancies, liquid biopsy approaches are increasingly being applied; however, their application in HCC remains limited. In this context, serum biomarkers represent a simple and cost-effective alternative [7,8]. Among the serum biomarkers, alpha-fetoprotein (AFP) and prothrombin induced by vitamin K absence-II (PIVKA-II), also known as des-γ-carboxy prothrombin (DCP), are the most widely investigated. AFP, the first HCC biomarker identified, is elevated in approximately half of patients but lacks specificity, as its levels may rise in chronic hepatitis or cirrhosis [7]. Elevated AFP levels correlate with poor differentiation, vascular invasion, and reduced survival; however, its prognostic utility remains inconsistent [9]. However, a substantial proportion of HCCs remain AFP-negative, particularly among early-stage tumors, supporting the need for complementary biomarkers such as PIVKA-II and Glypican-3 (GPC-3) [10,11].

PIVKA-II, an abnormal form of prothrombin produced by malignant hepatocytes, correlates with microvascular invasion, portal vein thrombosis, and early recurrence, reflecting a more aggressive tumor phenotype [12,13,14,15].

Early reports suggested that PIVKA-II might outperform AFP; however, their combined use enhanced diagnostic accuracy, forming the basis of composite scores such as GALAD [16]. Longitudinal studies have also shown that PIVKA-II displays greater temporal stability than AFP during surveillance of cirrhotic patients, whereas progressive increases in both biomarkers accompany HCC development, further supporting the biological relevance of PIVKA-II [17]. More recently, PIVKA-II-based models integrating routine clinical variables have been proposed to improve HCC risk stratification in patients with HCV-related cirrhosis after viral eradication, highlighting the expanding clinical applications of this biomarker [18]. Beyond its established diagnostic role, increasing interest has focused on the potential prognostic value of PIVKA-II. However, whether PIVKA-II provides independent prognostic information beyond conventional clinical staging, particularly in Western populations, remains unclear. The third biomarker, glypican-3 (GPC3), a cell-surface proteoglycan involved in cell proliferation and differentiation, is not hepatocyte-specific and can also be detected in non-hepatic malignancies such as cervical cancer, ovarian carcinoma, gastric cancer, and lung squamous carcinoma [19,20,21,22]. GPC3 is overexpressed in HCC tissues but rarely in normal hepatocytes [23]. Elevated serum GPC3 levels have been linked to tumor progression, reduced overall survival [24], and prognostic relevance in patients with HCC arising after HCV eradication, although its diagnostic performance remains less consistent [25]. Tissue overexpression of GPC-3 has been repeatedly linked to poorer prognosis and higher tumor aggressiveness in HCC; however, data on its serum levels and their prognostic implications are still scarce and inconclusive [26]. Recent evidence also suggests that circulating GPC-3 levels may reflect tumor GPC-3 expression and independently predict survival and treatment response in patients with advanced HCC treated with immunotherapy [27]. The three biomarkers differ substantially in their current clinical applicability, biological correlates and methodological limitations. Their main potential clinical roles and key diagnostic and prognostic limitations are summarized in Table 1.

Despite the inclusion of AFP in surveillance algorithms, none of the serum HCC biomarkers have yet been integrated into established prognostic models such as BCLC. Given the current shift in anti-cancer treatment towards biology-driven staging, a better understanding of the potential prognostic role of serum HCC biomarkers might improve prognostic stratification and treatment tailoring.

We assessed the prognostic value of AFP, PIVKA-II, and GPC-3 levels for overall survival in a large multicenter cohort of patients with newly diagnosed HCC.

## 2. Materials and Methods

### 2.1. Study Design and Population

We studied 605 consecutive patients newly diagnosed with HCC at two tertiary referral centers (Pisa and Turin) between January 2010 and January 2024. Inclusion criteria were age >18 years, HCC first diagnosed by imaging or histology according to the European Association for the Study of the Liver guidelines [2], and available measurements of AFP, PIVKA-II, and GPC-3 at baseline. The exclusion criteria were prior HCC diagnosis, BCLC-D stage, and follow-up <12 months for patients alive at the last follow-up. Patients who died within the first 12 months after HCC diagnosis were included in the analysis. The minimum 12-month follow-up requirement was applied only to patients alive at last observation to ensure an adequate observation period among censored individuals; patients who died within 12 months were retained because death represented a fully observed event.

The study was conducted in accordance with the principles of the Declaration of Helsinki and approved by our Institutional Ethics Committee.

For each patient, demographic, clinical, laboratory, and tumor-related variables were collected at the time of the first HCC diagnosis, including age, sex, etiology of liver disease, presence of cirrhosis and portal hypertension, ascites, Child-Pugh score, ALBI grade, MELD score, BCLC stage, portal vein tumor thrombosis, maximum diameter of HCC lesions, and serum levels of AFP, PIVKA-II, and GPC-3. The presence of cirrhosis was defined by histological and/or radiological findings (including liver surface nodularity, hepatic right lobe atrophy with caudate lobe hypertrophy, and parenchymal heterogeneity). Portal hypertension was diagnosed based on the presence of gastroesophageal varices, splenomegaly with thrombocytopenia (platelets <150.000/μL), and/or other indirect radiological signs, such as portosystemic collaterals or recanalization of the umbilical vein [28,29]. Following HCC diagnosis, patients were managed according to contemporary EASL recommendations and multidisciplinary tumor board decisions, with treatment allocation based on tumor stage, liver function, and patient performance status.

Overall survival (OS) was defined as the time (months) from HCC diagnosis to death from any cause or last follow-up.

### 2.2. Biomarkers Assessment

AFP was measured in fresh serum samples using the Pisa ARCHITECT chemiluminescent microparticle immunoassay (CMIA; Abbott Laboratories; Chicago, IL, USA; limit of detection 0.8 ng/mL, dynamic range, 0.8–22,451 ng/mL; upper normal limit 7.4 ng/mL); in Turin the fully automated chemiluminescent enzyme immunoassay (CLEIA) system Lumipulse^®^ G600 II (Fujirebio Inc., Tokyo, Japan) with Lumipulse^®^ G AFP-N reagent cartridges (limit of detection 0.075 ng/mL, dynamic range 0.5–2000 ng/mL, upper normal limit 7.4 ng/mL). PIVKA-II was measured by automated chemiluminescent enzyme immunoassays (Lumipulse G1220 or Lumipulse G600 platforms -Fujirebio Inc., Tokyo, Japan) using PIVKA-II reagent cartridges (detection limit 1.37 mAU/mL, dynamic range 1.37–75,000 mAU/mL, upper normal limit 48 mAU/mL). GPC-3 was measured at both centers using the same commercially available enzyme-linked immunosorbent assay (CanAg Glypican-3 EIA, Fujirebio Diagnostics AB, Gothenburg, Sweden; limit of detection 0 pg/mL, dynamic range 0–2710 pg/mL, upper normal limit 55 pg/mL).

### 2.3. Statistical Analysis

Continuous variables are expressed as median (range) and compared using the Mann–Whitney or Kruskal–Wallis test, as appropriate. Categorical variables are summarized as frequencies and compared using χ^2^ or Fisher’s exact test. AFP, PIVKA-II, and GPC-3 levels were analyzed as both continuous and categorical variables.

For categorical analyses, predefined prognostic thresholds, selected a priori on the basis of previously published literature, were used, specifically AFP >20 ng/mL, PIVKA-II >200 mAU/mL, and GPC-3 >150 pg/mL [24,30,31,32]. Survival curves were generated using the Kaplan–Meier method and compared using the log-rank test. Univariate and multivariate Cox proportional hazards models were fitted to assess the associations between variables and OS. Variables significantly associated with OS in univariate analysis were considered for inclusion in the multivariable model. To limit redundancy among partially overlapping measures of hepatic functional impairment, ALBI grade and ascites were retained in the primary multivariable model, whereas Child–Pugh class and MELD score were not simultaneously included. The degree of association between these liver-function measures was quantified using Spearman correlation analysis. As a sensitivity analysis, an additional fully adjusted model including Child–Pugh class and MELD score together with the variables of the primary model was fitted to assess the robustness of the biomarker estimates. Model discrimination was assessed using Harrell’s concordance index (C-index), and calibration was evaluated at 36 and 60 months. Internal validation was performed using 1000 bootstrap resamples to estimate optimism-corrected model performance, including the C-index and calibration slope. Calibration accuracy was additionally quantified using the mean absolute calibration error at each time point. The proportional hazards assumption was formally assessed using scaled Schoenfeld residuals. When evidence of non-proportionality was detected, additional stratified Cox models were fitted as sensitivity analyses to assess the robustness of the biomarker estimates. Statistical significance was defined as a two-sided *p*-value of <0.05. All analyses were performed using JMP Pro 17 software (version 17.0.1, SAS Institute Inc., Cary, NC, USA) and R software (version 4.5.1)

## 3. Results

### 3.1. Patient Characteristics and Cohort Overview

Baseline demographic, clinical, HCC staging, and tumor marker levels of the entire cohort are reported in Table 2; a detailed comparison of baseline characteristics between the Pisa and Turin cohorts is provided in Supplementary Table S1. The median age at HCC diagnosis was 68.8 years (range 32.6–91.4), and 463 (76.5%) patients were male. The underlying liver disease was HCV-related in 316 patients (52.2%), metabolic-associated steatotic liver disease (MASLD) in 113 (18.7%), cirrhosis in 552 (91.2%), and ascites in 115 (19.1%). Most patients had well-preserved hepatic function, with 490 (80.9%) classified as Child–Pugh A, 321 (53.1%) as ALBI grade 1, and portal hypertension was diagnosed in 253 (41.8%) cases. Portal vein tumor thrombosis was detected in 56 (9.3%) patients in the overall cohort. According to BCLC staging, 83 (13.7%) of patients were classified as stage BCLC-0, 284 (46.9%) as stage A, 151 (24.9%) as stage B, and 87 (14.4%) as stage C. Median serum levels of AFP, PIVKA-II, and GPC-3 were 7.9 ng/mL (range 0.8–87,564), 179 mAU/mL (range 9–597,830), and 85.6 pg/mL (range 0.1–7260), respectively. The AFP level was >20 ng/mL in 201 (33.2%) patients, PIVKA-II >200 mAU/mL in 288 (47.6%), and GPC-3 >150 pg/mL in 177 (29.3%) patients. The median overall survival (OS) was 29.0 months (range 0.1–176.6), and at the last follow-up, 261 (43.1%) patients were alive.

### 3.2. Correlation Between Tumor Markers and Clinical Variables

To better characterize each tumor marker profile, pairwise correlations were explored between the three serum biomarkers and the main clinical and tumor-related variables. Weak but statistically significant correlations were observed between the biomarkers themselves: AFP and PIVKA-II (ρ = 0.28, *p* < 0.001), PIVKA-II and GPC-3 (ρ = 0.22, *p* < 0.001), and a moderate correlation between AFP and GPC-3 (ρ = 0.43, *p* < 0.001). None of the biomarkers were significantly associated with age. PIVKA-II was weakly but significantly correlated with the MELD score (ρ = 0.12, *p* = 0.004), whereas no significant associations were observed between tumor markers and ALBI scores. All tumor markers showed modest positive correlations with maximum lesion diameter (AFP ρ = 0.23; PIVKA-II ρ = 0.50; GPC-3 ρ = 0.30; all *p* < 0.001) and a weak inverse correlation with overall survival (AFP ρ = −0.13; PIVKA-II ρ = −0.15; GPC-3 ρ = −0.15; all *p* < 0.001). All the correlation coefficients are reported in Supplementary Table S2.

### 3.3. Clinical and Tumor Marker Characteristics Across BCLC Stages

Detailed demographic, clinicopathological, and tumor marker data according to BCLC stage are reported in Supplementary Table S3, whereas Figure 1 shows tumor marker levels across the BCLC stages. Serum biomarker levels above the predefined thresholds increased progressively across BCLC stages for all three biomarkers, although the trend was most pronounced for PIVKA-II. In BCLC-0, only 18.1% of the patients had PIVKA-II levels >200 mAU/mL, compared with 38.4% in BCLC-A, 62.9% in BCLC-B, and 79.3% in BCLC-C. Likewise, the median PIVKA-II values increased progressively across stages, reaching 1397 mAU/mL in BCLC-C (Figure 1; *p* < 0.001). AFP and GPC-3 levels also increased with advancing BCLC stage, although less consistently than those of PIVKA-II. (Figure 1; *p* < 0.001).

### 3.4. Univariable Survival Analysis

Survival progressively decreased across BCLC stages (*p* < 0.001, Table S3). Univariate Cox regression OS was associated with cirrhosis, ascites, portal hypertension, portal vein thrombosis, Child-Pugh score, MELD score, and ALBI score (higher values were associated with worse outcomes, *p* < 0.001 for all). Elevated AFP, PIVKA-II, and GPC-3 levels were significantly associated with shorter OS (all *p* < 0.001). When analyzed as categorical variables (AFP > 20 ng/mL, PIVKA-II > 200 mAU/mL, and GPC-3 > 150 pg/mL), values above the cutoff values consistently identified subgroups with significantly shorter survival (all *p* < 0.001). Age at diagnosis, sex, and etiology were not significantly associated with OS. Univariable analyses and Kaplan–Meier survival curves according to tumor marker cut-offs are reported in Supplementary Table S4 and Figure S1.

### 3.5. Multivariable Survival Analysis

A multivariable Cox proportional hazards model (Table 3, Figure 2) was used, including variables that were statistically significant in the univariate analysis, after accounting for overlap among measures of liver function. Specifically, we included ascites and ALBI grade, whereas Child–Pugh class and MELD score were excluded. Particularly, Child–Pugh class was associated with ALBI grade (Spearman ρ = 0.47, *p* < 0.001) and ascites (ρ = 0.56, *p* < 0.001), whereas MELD score was associated with ALBI grade (ρ = 0.43, *p* < 0.001) and ascites (ρ = 0.30, *p* < 0.001). The model also included BCLC stage, and the three serum tumor markers (AFP, PIVKA-II, and GPC-3) were entered as categorical variables according to clinically relevant cut-off values. The multivariable analysis showed that OS was independently associated with tumor stage (BCLC-B vs. 0: HR 1.59, 95% CI 1.02–2.49; BCLC-C vs. 0: HR 3.03, 95% CI 1.86–4.94) (ALBI grade 2–3: HR 1.36, 95% CI 1.08–1.71), ascites (HR 2.19, 95% CI 1.67–2.87), and PIVKA-II >200 mAU/mL (HR 1.56, 95% CI 1.24–1.95), with a trend for GPC-3 >150 pg/mL (HR 1.27, 95% CI 0.99–1.63). Compared with BCLC-0, BCLC-A did not significantly affect the risk of death (HR 0.96, 95% CI 0.63–1.48; *p* = 0.882), whereas BCLC-B was associated with a 59% higher risk (HR 1.59, 95% CI 1.02–2.49; *p* = 0.042), and BCLC-C nearly tripled the mortality risk (HR 3.03, 95% CI 1.86–4.94; *p* < 0.001). The presence of ascites remained an independent adverse prognostic factor (HR 2.19, 95% CI 1.67–2.87; *p* < 0.001), and an ALBI grade of 2–3 was associated with a 36% higher mortality compared with grade 1 (HR 1.36, 95% CI 1.08–1.71; *p* = 0.009). In a sensitivity model additionally including Child–Pugh class and MELD score, the association between PIVKA-II >200 mAU/mL and OS remained consistent (HR 1.40, 95% CI 1.11–1.76; *p* = 0.005). The primary multivariable model showed moderate discrimination, with an apparent Harrell’s C-index of 0.701 (95% CI 0.671–0.731). After 1000 bootstrap resamples, the optimism-corrected C-index was 0.695 and the optimism-corrected calibration slope was 0.942. Mean absolute calibration error was 0.044 at 36 months and 0.076 at 60 months, with higher calibration error at the longer prediction horizon.

Formal assessment of the proportional hazards assumption showed no evidence of non-proportionality for BCLC stage, AFP, or PIVKA-II, whereas deviations were observed for ALBI grade, ascites, and GPC-3. The global Schoenfeld test was therefore significant (*p* = 0.004; Supplementary Table S5a). Sensitivity analyses accounting for the non-proportional covariates confirmed the association between PIVKA-II and OS (Supplementary Table S5b).

## 4. Discussion

The three circulating biomarkers investigated in this study, AFP, PIVKA-II and GPC-3, showed distinct patterns of association with tumor characteristics and OS. Serum marker levels were associated with tumor burden, with PIVKA-II showing the strongest correlation with HCC diameter (ρ = 0.50, *p* < 0.001), compared with GPC-3 (ρ = 0.30, *p* < 0.001) and AFP (ρ = 0.23, *p* < 0.001). Conversely, the tumor marker relationship with liver function appeared irrelevant: absent for all three markers when considering ALBI score and weakly significant only for PIVKA-II with MELD score (ρ = 0.12, *p* = 0.004). After adjustment for BCLC stage and liver function. After adjustment for BCLC stage and liver function, PIVKA-II remained independently associated with OS (HR: 1.56; *p* < 0.001), whereas GPC-3 showed a borderline association (HR:1.27; *p* = 0.058), and AFP lost its prognostic significance (*p* = 0.306).

Our findings agree with those of previous studies, suggesting that circulating biomarkers primarily reflect tumor biological aggressiveness, thereby providing prognostic information complementary to conventional HCC staging and liver function assessment. Therefore, tumor marker-based prognosis does not appear redundant with the combined liver function and tumor staging of the BCLC system, adding a tumor-specific prognostic component.

In univariate analyses, all three tumor markers showed significant inverse correlations with OS both as continuous variables (AFP ρ = −0.13, PIVKA-II ρ = −0.15, GPC-3 ρ = −0.15; all *p* < 0.001) and when dichotomized at predefined cut-offs (AFP >20 ng/mL, PIVKA-II >200 mAU/mL, GPC-3 >150 pg/mL), with PIVKA-II showing the clearest survival separation in the Kaplan–Meier analysis based on the 200 mAU/mL cut-off (Supplementary Figure S1). Multivariable analysis showed different patterns of association with OS across the three biomarkers. After adjustment for tumor stage and liver function, PIVKA-II remained independently associated with shorter survival, whereas GPC-3 showed a borderline association and AFP was no longer independently associated with OS. These findings do not necessarily imply a lack of biological relevance for GPC-3 and/or AFP but rather emphasize that each biomarker captures different biological features of HCC with different degrees of independence from the already established clinical prognostic variables. AFP, PIVKA-II and GPC-3 showed statistically significant but weak/moderate correlations: AFP with PIVKA-II and GPC-3, for both *p* < 0.001, but ρ = 0.28 and 0.43 respectively; PIVKA-II and GPC-3 ρ = 0.22, *p* < 0.001.

The prognostic role of AFP in HCC remains heterogeneous across studies. Elevated baseline AFP has been associated with adverse tumor features and poorer outcomes in several clinical cohorts [31,32], whereas other studies, particularly in patients with small HCC detected during surveillance and preserved liver function, have not demonstrated an independent prognostic effect [9]. These discrepancies may reflect differences in tumor stage, underlying liver disease, hepatic inflammatory activity, treatment setting, AFP thresholds, and the clinical variables included in multivariable adjustment. Indeed, AFP is not exclusively related to tumor biology, as its serum levels may also increase in the setting of hepatic inflammation and regeneration [33,34]. Conversely, AFP elevation in the absence of active liver injury may more specifically reflect neoplastic transformation [35,36]. In our cohort, AFP was associated with tumor burden and with shorter survival in univariable analysis, but this association disappeared after adjustment for established clinical prognostic factors. Therefore, our findings should not be interpreted as indicating an absence of prognostic relevance of AFP in HCC overall, but rather as suggesting limited independent prognostic information from baseline AFP within the multivariable setting evaluated in the present cohort.

GPC-3, a membrane-bound heparan sulfate proteoglycan involved in the regulation of cell proliferation through the Wnt/β-catenin and Hedgehog signaling pathways, has emerged as a promising biomarker for HCC [37]. Tissue GPC-3 overexpression has consistently been associated with poor prognosis and aggressive tumor behavior; however, evidence regarding the prognostic value of circulating GPC-3 remains limited and inconclusive [24,38,39]. The clinical interpretation of circulating GPC-3 is further limited by heterogeneity and lack of standardization in assay methodology and proposed cut-offs, with no widely accepted standardized threshold. In the present study, this source of variability was minimized by using the same commercially available assay at both participating centers. In our cohort, GPC-3 levels increased progressively across BCLC stages and were correlated with tumor burden, supporting its potential role as a marker of HCC aggressiveness. Interestingly, GPC-3 showed a borderline independent association with OS, suggesting the need for future studies with larger cohorts to better define its role in prognostication.

PIVKA-II, an abnormal prothrombin produced by HCC cells due to defective vitamin K–dependent γ-carboxylation, has been implicated in several mechanisms promoting HCC progression, as it promotes tumor growth by stimulating vascular endothelial growth factor (VEGF) and activation of the Met/JAK–STAT3 signaling pathway [40]. Accordingly, elevated serum PIVKA-II levels have been associated with microvascular invasion, tumor recurrence, and poor overall survival [10,11,12,13,14,15,41,42,43]. However, interpretation of PIVKA-II should account for potential non-malignant confounders. Serum levels may be influenced by disturbances in vitamin K metabolism, including vitamin K antagonist therapy and cholestatic conditions, resulting in elevations unrelated to HCC [44].

Beyond its prognostic value at diagnosis, longitudinal modelling of PIVKA-II kinetics during systemic treatment has suggested that changes in PIVKA-II may reflect tumor biological behavior and treatment response, supporting its potential role as a dynamic biomarker in HCC [45]. However, changes in serum biomarkers may not necessarily parallel radiological response, as serological and imaging assessments may capture different aspects of tumor response. Integrating biomarker kinetics with imaging may therefore better characterize heterogeneous treatment trajectories during systemic therapy [45]. Accordingly, longitudinal biomarker assessment should be considered complementary to, rather than a substitute for, imaging-based response evaluation. In our cohort, we showed not only a strong correlation between PIVKA-II and tumor diameter but also its independent prognostic value when considering patient survival. However, whether patients with elevated PIVKA-II may benefit from biomarker-informed risk stratification or management strategies remains to be determined prospectively.

These findings were obtained in a cohort whose clinic–demographic characteristics were consistent with previous reports from Western countries, comprising predominantly older male patients with HCV-related cirrhosis and a balanced distribution across early and intermediate BCLC stages [2,46,47]. Importantly, our findings agree with those of previous reports obtained mainly in Asian HBV-prevalent populations, extending the prognostic value of PIVKA-II to a Western cohort with predominantly HCV-related liver disease.

Overall, our findings support the potential value of incorporating circulating tumor markers into prognostic assessment at the time of HCC diagnosis. Patients with PIVKA-II levels >200 mAU/mL showed poorer survival independently of tumor stage and liver function. This higher-risk subgroup warrants prospective evaluation to determine whether biomarker-guided risk stratification and management strategies may translate into clinical benefit [48,49].

Our study has several limitations. First, the retrospective design may be associated with selection bias and residual confounding. Although consecutive patients with newly diagnosed HCC were included, eligibility required the availability of baseline measurements of all three biomarkers, which may have selected patients with more complete clinical and laboratory characterization. Second, the extended inclusion period (2010–2024) encompassed major changes in HCC management, including the introduction of novel locoregional and systemic treatments. Because treatment exposure was not standardized across the study period, residual confounding related to treatment era cannot be excluded when interpreting overall survival. Third, although the study was multicenter, both participating institutions were tertiary referral centers in Italy, and the cohort was predominantly representative of a Western HCC population. Therefore, the generalizability of our findings to populations with different geographic and etiological backgrounds requires external validation. Finally, although the biomarker cut-offs were predefined from the previous literature and were not optimized within the present dataset, dichotomization inevitably reduces biological and statistical information and may limit reproducibility across populations and assay settings. Analyses considering biomarker levels as continuous variables therefore provide complementary information.

## 5. Conclusions

In this multicenter retrospective cohort, PIVKA-II was independently associated with OS after adjustment for BCLC stage and liver function, whereas GPC-3 showed a borderline association and AFP was not independently associated with OS. Overall, these findings support further investigation of PIVKA-II as a complementary prognostic biomarker in HCC. Prospective external validation and formal assessment of its incremental predictive value are required before integration into established prognostic models can be recommended.

## Figures and Tables

**Figure 1:**
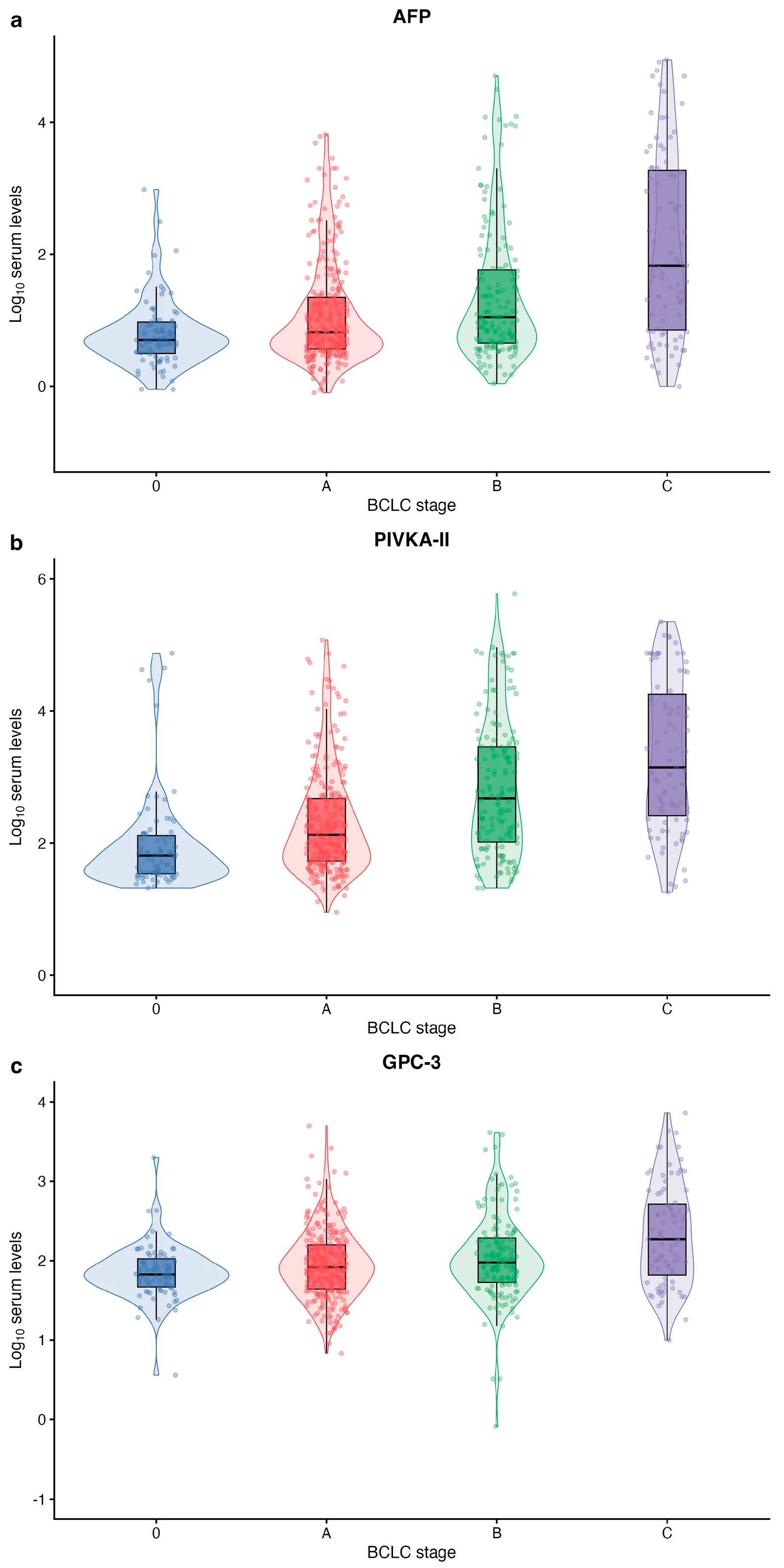
Serum tumor marker levels according to BCLC stage. Violin plots with superimposed boxplots and individual observations show the distribution of log10-transformed serum AFP (**a**), PIVKA-II (**b**), and GPC-3 (**c**) levels across BCLC stages 0–C. Colors indicate BCLC stage: stage 0, blue; stage A, red; stage B, green; and stage C, purple.

**Figure 2:**
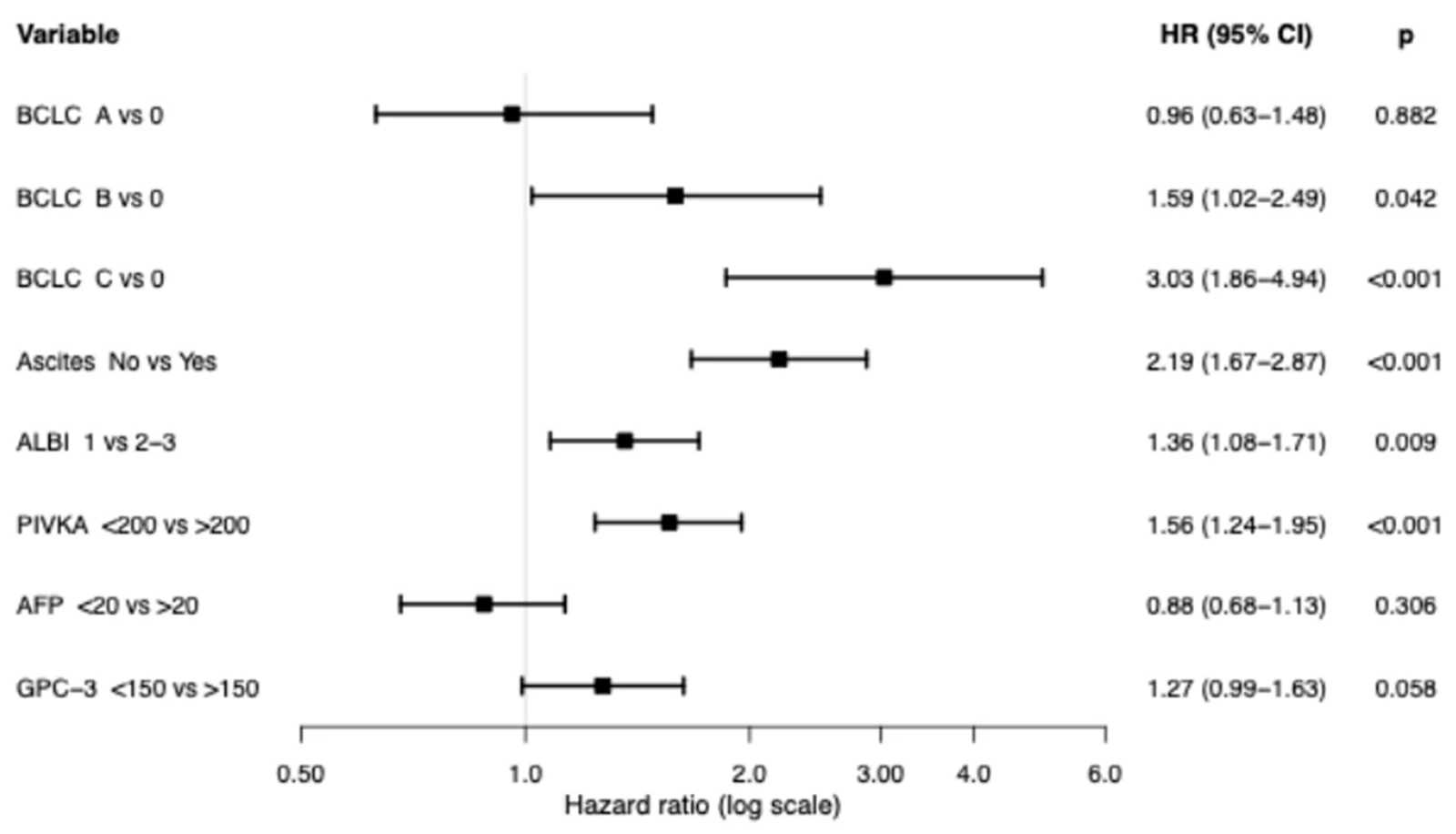
Forest plot of multivariable Cox regression analysis for overall survival. Legend: Hazard ratios (HRs) and 95% confidence intervals (CIs) are shown. The vertical line indicates HR = 1. BCLC stage 0 was used as the reference category. BCLC, Barcelona Clinic Liver Cancer; ALBI, albumin–bilirubin score; AFP, alpha-fetoprotein; PIVKA-II, protein induced by vitamin K absence or antagonist-II; GPC-3, glypican-3.

**Table 1:** Main clinical applications and limitations of AFP, PIVKA-II, and GPC-3 in hepatocellular carcinoma.

Biomarker	Main Clinical Value in HCC	Main Limitations
AFP	Most widely used serum biomarker in HCC. Elevated levels may reflect tumor burden and adverse biological features and can provide prognostic information in selected clinical settings [7,9].	Limited sensitivity (because of AFP-negative HCC and in early-stage HCC); limited specificity because serum levels may also increase in presence of necro-inflammation and regeneration. Prognostic performance is inconsistent across clinical settings [7,9,10,11].
PIVKA-II	Associated with tumor aggressiveness, microvascular invasion, recurrence and adverse prognosis. It provides complementary information to AFP and is incorporated into composite biomarker models such as GALAD [10,13,16,17,18].	Not yet incorporated into established prognostic frameworks such as BCLC, and its independent prognostic value beyond conventional staging remains incompletely defined, particularly in Western populations [10,12,13,14,15,16,17,18].
GPC-3	Well-established tissue marker of HCC; circulating GPC-3 has also been investigated as a serum biomarker for diagnosis and prognosis, with higher levels associated with tumor progression and adverse outcomes [11,23,24,25,26,27].	Circulating GPC-3 is not entirely tumor- or organ-specific, and serum diagnostic and prognostic performance remains heterogeneous and less well established than tissue GPC-3 [19,20,21,22,23,24,25,26].

AFP, alpha-fetoprotein; PIVKA-II, protein induced by vitamin K absence or antagonist-II; GPC-3, glypican-3.

**Table 2:** Baseline demographics, clinic–pathologic characteristics and tumor markers.

Variable		Overall (*n* = 605)
Age	Years	68.8 (32.6–91.4)
Sex	Male	463 (76.5)
	Female	142 (23.5)
Etiology	HCV	316 (52.2)
	HBV/HDV	71 (11.7)
	MASLD	113 (18.7)
	Alcool	85 (14.1)
	Other	20 (3.3)
OS	Months	29.0 (0.1–176.6)
Status at last Observation	Alive	261 (43.1)
	Death	344 (56.9)
Cirrhosis	No	53 (8.8)
	Yes	552 (91.2)
Ascites	No	490 (80.9)
	Yes	115 (19.1)
Child-Pugh Class	A	490 (80.9)
	B-C	115 (19.1)
ALBI grade	1	321 (53.1)
	2–3	284 (46.9)
MELD		9 (6–27)
Portal Hypertension	No	352 (58.2)
	Yes	253 (41.8)
BCLC	0	83 (13.7)
	A	284 (46.9)
	B	151 (24.9)
	C	87 (14.4)
Portal Vein Thrombosis	No	549 (90.7)
	Yes	56 (9.3)
AFP	ng/mL	7.9 (0.8–87,564)
AFP	<20 ng/mL	404 (66.8)
	>20 ng/mL	201 (33.2)
PIVKA-II	mAU/mL	179 (9–597,830)
PIVKA-II	<200 mAU/mL	317 (52.4)
	>200 mAU/mL	288 (47.6)
GPC-3	pg/mL	85.6 (0.1–7260.1)
GPC-3	<150 pg/mL	428 (70.7)
	>150 pg/mL	177 (29.3)

Data are expressed as median (range) or number (percentage). Legend: HCV, hepatitis C virus; HBV, hepatitis B virus; HDV, hepatitis D virus; MASLD, metabolic-associated steatotic liver disease; OS, overall survival; ALBI, albumin–bilirubin grade; MELD, Model for End-Stage Liver Disease; BCLC, Barcelona Clinic Liver Cancer; AFP, alpha-fetoprotein; PIVKA-II, protein induced by vitamin K absence or antagonist-II; GPC-3, glypican-3.

**Table 3:** Multivariable Cox regression analysis for overall survival.

Variable		HR	95% CI	*p*-Value
BCLC	A vs. 0	0.96	0.63–1.48	0.882
	B vs. 0	1.59	1.02–2.49	**0.042**
	C vs. 0	3.03	1.86–4.94	**<0.001**
Ascites	Yes vs. No	2.19	1.67–2.87	**<0.001**
ALBI	2–3 vs. 1	1.36	1.08–1.71	**0.009**
PIVKA-II	> 200 mAU/mL	1.56	1.24–1.95	**<0.001**
AFP	>20 ng/mL	0.88	0.68–1.13	0.306
GPC-3	>150 pg/mL	1.27	0.99–1.63	0.058

Legend: HR, hazard ratio; CI, confidence interval; BCLC, Barcelona Clinic Liver Cancer; ALBI, albumin–bilirubin score; AFP, alpha-fetoprotein; PIVKA-II, protein induced by vitamin K absence or antagonist-II; GPC-3, glypican-3.

## Data Availability

The data that support the findings of this study are available from the corresponding author upon reasonable request. The data are not publicly available due to privacy and ethical restrictions related to patient-level clinical data.

## Supplementary Materials

The following supporting information can be downloaded at: https://www.mdpi.com/article/10.3390/biomedicines14102146/s1, Figure S1. Kaplan–Meier estimates of overall survival according to baseline AFP (a), PIVKA-II (b), and GPC-3 (c) levels; Table S1. Comparison of baseline clinical, tumor-related, and biochemical characteristics between the Pisa and Turin cohorts; Table S2. Correlations between circulating biomarkers, clinical and tumor-related variables, and overall survival; Table S3. Clinical and biomarker characteristics across BCLC stages; Table S4. Univariate Cox regression analysis for overall survival; Table S5a. Proportional hazards assumption in the primary multivariable Cox model; Table S5b. Multivariable Cox sensitivity analysis stratified by ALBI grade, ascites, and GPC-3.

## Abbreviations

The following abbreviations are used in this manuscript:

AFP	Alpha-fetoprotein
ALBI	Albumin–bilirubin
BCLC	Barcelona Clinic Liver Cancer
CI	Confidence interval
CLEIA	Chemiluminescent enzyme immunoassay
CMIA	Chemiluminescent microparticle immunoassay
DCP	Des-γ-carboxy prothrombin
EIA	Enzyme immunoassay
GPC-3	Glypican-3
HBV	Hepatitis B virus
HCC	Hepatocellular carcinoma
HCV	Hepatitis C virus
HDV	Hepatitis D virus
HR	Hazard ratio
MASLD	Metabolic dysfunction-associated steatotic liver disease
MELD	Model for End-Stage Liver Disease
OS	Overall survival
PIVKA-II	Protein induced by vitamin K absence-II
VEGF	Vascular endothelial growth factor
