# Supplementary material for: Comparative Prognostic Value of AFP, PIVKA-II, and GPC-3 in Hepatocellular Carcinoma: A Real-World Multicenter Cohort Study
Source: Biomedicines. 2026 Sep 23;14(10):2146. doi: 10.3390/biomedicines14102146 (PMC13701469; doi:10.3390/biomedicines14102146)
Supplement: Supplementary file 1 [file biomedicines-14-02146-s001.zip › biomedicines-4552049-supplementary.pdf]

SUPPLEMENTARY MATERIALS

		Pisa (n =279)	Turin (n =326)	P-value
Age		73.0 (32.6-91.4)	65.2 (44.2-87.5)	<0.001
Sex	Male	207 (74.2)	256 (78.5)	0.178
	Female	72 (25.8)	70 (21.5)	
Etiology	HCV	125 (44.8)	191 (58.6)	<0.001
	HBV/HDV	31 (11.1)	40 (12.3)	
	MASLD	80 (28.7)	33 (10.1)	
	Alcool	37 (13.3)	48 (14.7)	
	Other	6 (2.1)	14 (4.3)	
OS	Months	32.2 (1.1-176.6)	28.4 (0.1-129.5)	0.024
Status at last Observation	Alive	137 (49.1)	124 (38.0)	0.006
	Death	142 (50.9)	202 (62.0)	
Cirrhosis	No	18 (6.5)	35 (10.7)	0.061
	Yes	261 (93.5)	291 (89.3)	
Ascites	No	245 (87.8)	245 (75.1)	<0.001
	Yes	34 (12.2)	81 (24.9)	
Child-Pugh Class	A	256 (91.8)	234 (71.8)	<0.001
	B-C	23 (8.2)	92 (28.2)	
ALBI grade	1	203 (72.3)	118 (36.2)	<0.001
	2-3	76 (27.2)	208 (63.8)	
MELD		9 (6-22)	10 (6-27)	<0.001
Portal Hypertension	No	202 (72.4)	150 (46.0)	<0.001
	Yes	77 (27.6)	176 (54.0)	
BCLC	0	44 (15.7)	39(12.0)	0.003
	A	109 (39.0)	175 (53.7)	
	B	77 (27.6)	74 (22.7)	
	C	49 (17.6)	38 (11.6)	
Portal Vein Thrombosis	No	252 (89.9)	297 (91.1)	0.633
	Yes	27 (10.0)	29 (8.9)	
AFP	ng/ml	6.4 (0.8-87564)	11.2 (0.9-80961)	0.003
AFP	<20	206 (73.8)	198 (60.7)	<0.001
	>20	73 (26.2)	128 (39.3)	
PIVKA	mAU/ml	177 (13-597830)	179 (9-75000)	0.313
PIVKA	<200	144 (51.6)	173 (53.1)	0.721
	>200	135 (48.4)	153 (46.9)	

GPC-3	pg/ml	80.6<br>(0.0-7260.0)	98.5<br>(9-4981)	0.013
GPC-3	<150	202 (72.4)	226 (69.3)	0.407
	>150	77 (27.6)	100 (30.7)	

Supplementary Table S1. Comparison of baseline clinical, tumor-related, and biochemical characteristics between the Pisa and Turin cohorts.
Legend: Baseline characteristics were compared between patients enrolled at the Pisa and Turin centers. Continuous variables are reported as median (range) and categorical variables as number (percentage). BCLC, Barcelona Clinic Liver Cancer; ALBI, albumin–bilirubin score; AFP, alpha-fetoprotein; PIVKA-II, protein induced by vitamin K absence or antagonist-II; GPC-3, glypican-3.

		**AFP**	**PIVKA**	**GPC-3**
**AFP**	ρ<br>P	/<br>/	0.280<br><0.001	0.431<br><0.001
**PIVKA**	ρ<br>P	0.280<br><0.001	/<br>/	0.223<br><0.001
**GPC-3**	ρ<br>P	0.431<br><0.001	0.223<br><0.001	/<br>/
**Age at Diagnosis**	ρ<br>P	-0.069<br>0.088	-0.031<br>0.449	-0.018<br>0.661
**Max lesion diameter**	ρ<br>P	0.230<br><0.001	0.498<br><0.001	0.302<br><0.001
**MELD Score**	ρ<br>P	0.001<br>0.979	0.124<br><0.001	0.005<br>0.910
**ALBI Score**	ρ<br>P	0.001<br>0.978	0.028<br>0.503	0.061<br>0.132
**Overall Survival**	ρ<br>P	-0.129<br><0.001	-0.150<br><0.001	-0.149<br><0.001

Supplementary Table S2. Correlations between circulating biomarkers, clinical and tumor-related variables, and overall survival.
Legend: Correlations were assessed between biomarker levels and clinical, tumor-related, and survival-related variables. BCLC, Barcelona Clinic Liver Cancer; ALBI, albumin–bilirubin score; AFP, alpha-fetoprotein; PIVKA-II, protein induced by vitamin K absence or antagonist-II; GPC-3, glypican-3.

		BCLC-0	BCLC-A	BCLC-B	BCLC-C	P
Age		70.8 (44.2-87.3)	69.0 (44.2-90.3)	68.8 (43.7-91.5)	68.8 (32.6-87.5)	0.438
Sex	Male	52 (62.6)	210 (74.2)	126 (83.4)	75 (86.2)	<0.001
	Female	31 (37.4)	74 (25.8)	25 (16.6)	12 (13.8)	
Etiology	HCV	48 (57.8)	165 (58.1)	64 (42.4)	39 (44.8)	0.077
	HBV/HDV	10 (12.1)	30 (10.6)	17 (11.3)	14 (16.1)	
	MASLD	9 (10.8)	46 (16.2)	39 (25.9)	19 (21.8)	
	Alcol	13 (15.7)	34 (11.9)	27 (17.9)	11 (12.6)	
	Other	3 (3.6)	9 (3.2)	4 (2.7)	4 (4.6)	
OS	Months	47.9 (0.1-114.8)	34.2 (0.7-176.6)	23.2 (0.9-122.5)	10.7 (0.5-94.0)	<0.001
Status at last OBV	Alive	56 (67.5)	131 (46.1)	53 (35.1)	21 (24.1)	<0.001
	Death	27 (32.5)	153 (53.9)	98 (64.9)	66 (75.9)	
Cirrhosis	No	7 (8.4)	28 (9.9)	16 (10.6)	2 (2.3)	0.063
	Yes	76 (91.6)	256 (90.1)	135 (89.4)	85 (97.7)	
Ascites	No	77 (92.8)	231 (81.3)	120 (79.5)	62 (71.3)	<0.001
	Yes	6 (7.2)	53 (18.7)	31 (20.6)	25 (28.7)	
Child-Pugh Class	A	79 (95.2)	228 (80.3)	118 (78.2)	65 (74.7)	<0.001
	B-C	4 (4.8)	56 (19.7)	33 (21.9)	22 (25.3)	
ALBI grade	1	59 (71.1)	142 (50.0)	85 (56.3)	35 (40.2)	<0.001
	2-3	24 (28.9)	142 (50.0)	66 (43.7)	52 (59.8)	
MELD		9 (6-16)	10 (6-27)	9 (6-25)	9 (6-21)	0.239
Portal Hypertension	No	53 (63.9)	165 (58.1)	91 (60.3)	43 (49.4)	0.252
	Yes	30 (36.1)	119 (41.9)	60 (39.7)	44 (50.6)	
AFP		5 (0.9-950.6)	6.6 (0.8-6507)	11.1 (1.1-50000)	66.8 (1-14947)	<0.001
AFP	<20	73 (87.9)	210 (73.9)	92 (60.9)	29 (33.3)	<0.001
	>20	10 (12.1)	74 (26.1)	59 (39.1)	58 (66.7)	
PIVKA		65 (21-75000)	135 (9-119530)	476 (21-597830)	1397 (18-226140)	<0.001
PIVKA	<200	68 (81.9)	175 (61.6)	56 (37.1)	18 (20.7)	<0.001
	>200	15 (18.1)	109 (38.4)	95 (62.9)	69 (79.3)	
GPC-3		65.9 (0.0-1988.5)	82.5 (6.8-4981)	91.9 (0.0-4090)	186 (9.9-7260)	<0.001
GPC-3	<150	76 (91.6)	209 (73.6)	104 (68.9)	39 (44.8)	<0.001
	>150	7 (8.4)	75 (26.4)	47 (31.3)	48 (55.2)	

Supplementary Table S3. Clinical and biomarker characteristics across BCLC stages. Data are expressed as median (range) or number (percentage). Legend: *BCLC, Barcelona*

Clinic Liver Cancer; HCV, hepatitis C virus; HBV, hepatitis B virus; HDV, hepatitis D virus; MASLD, metabolic-associated steatotic liver disease; OS, overall survival; ALBI, albumin–bilirubin grade; MELD, Model for End-Stage Liver Disease; AFP, alpha-fetoprotein; PIVKA-II, protein induced by vitamin-K absence or antagonist-II; GPC-3, glypican-3.

		P-value
Age		0.844
Sex	Male	0.105
	Female	
Etiology	HCV	0.399
	HBV/HDV	
	MASLD	
	Alcool	
	Other	
Cirrhosis	No	<0.001
	Yes	
Ascites	No	<0.001
	Yes	
Child-Pugh Class	A	<0.001
	B-C	
ALBI grade	1	<0.001
	2-3	
MELD		<0.001
Portal Hypertension	No	<0.001
	Yes	
BCLC	0	<0.001
	A	
	B	
	C	
Portal Vein Thrombosis	No	<0.001
	Yes	
AFP	ng/ml	<0.001
AFP	<20	<0.001
	>20	
PIVKA	mAU/ml	<0.001
PIVKA	<200	<0.001
	>200	
GPC-3	pg/ml	<0.001
GPC-3	<150	<0.001
	>150	

Supplementary Table S4. Univariate Cox regression analysis for overall survival.
Legend: BCLC, Barcelona Clinic Liver Cancer; ALBI, albumin–bilirubin score; AFP, alpha-

fetoprotein; PIVKA-II, protein induced by vitamin K absence or antagonist-II; GPC-3, glypican-3.

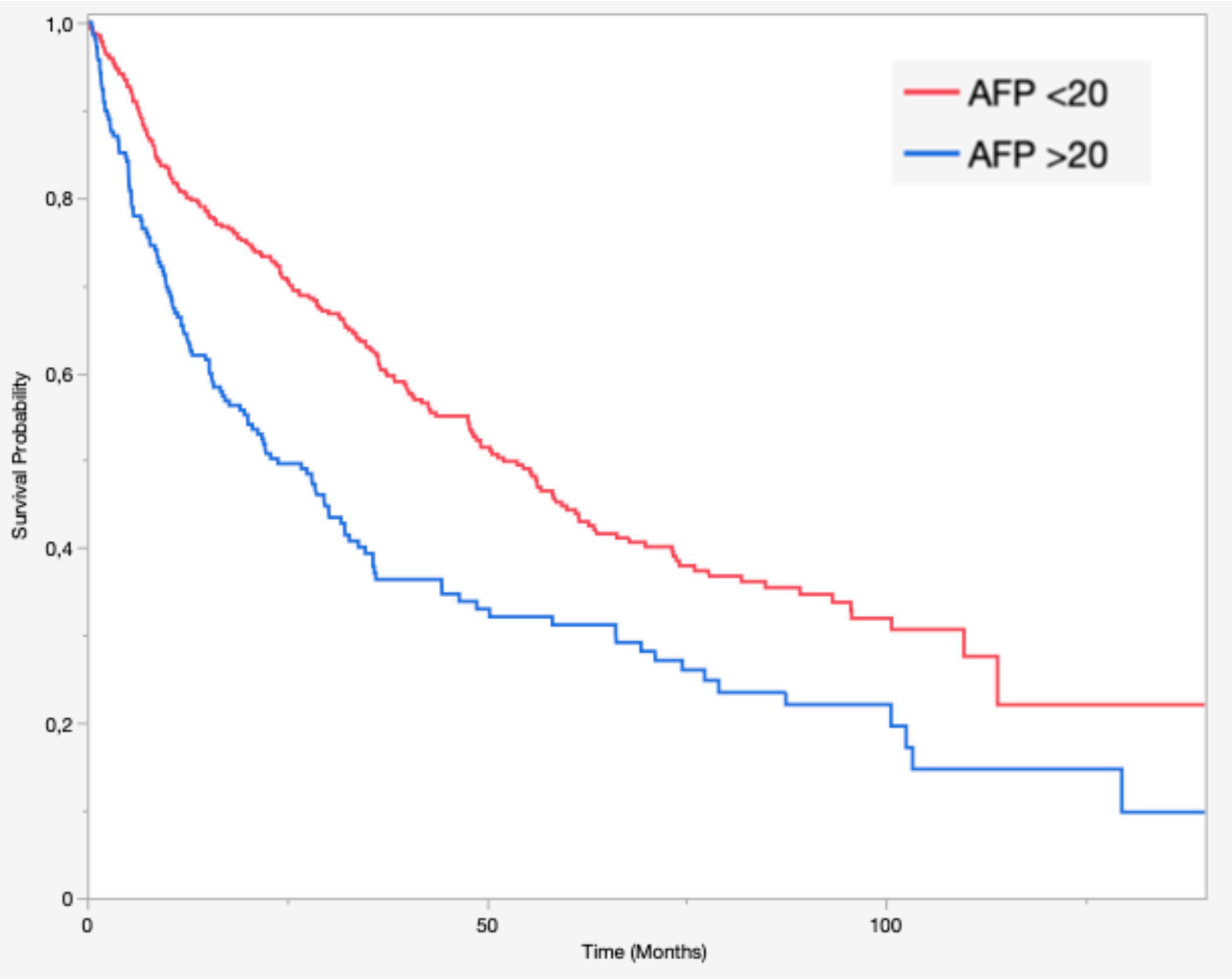

Figure S1a. Kaplan–Meier estimates of overall survival according to baseline AFP levels. Patients were stratified by AFP <20 ng/mL and ≥20 ng/mL. Time is expressed in months. Survival probabilities were estimated using the Kaplan–Meier method.

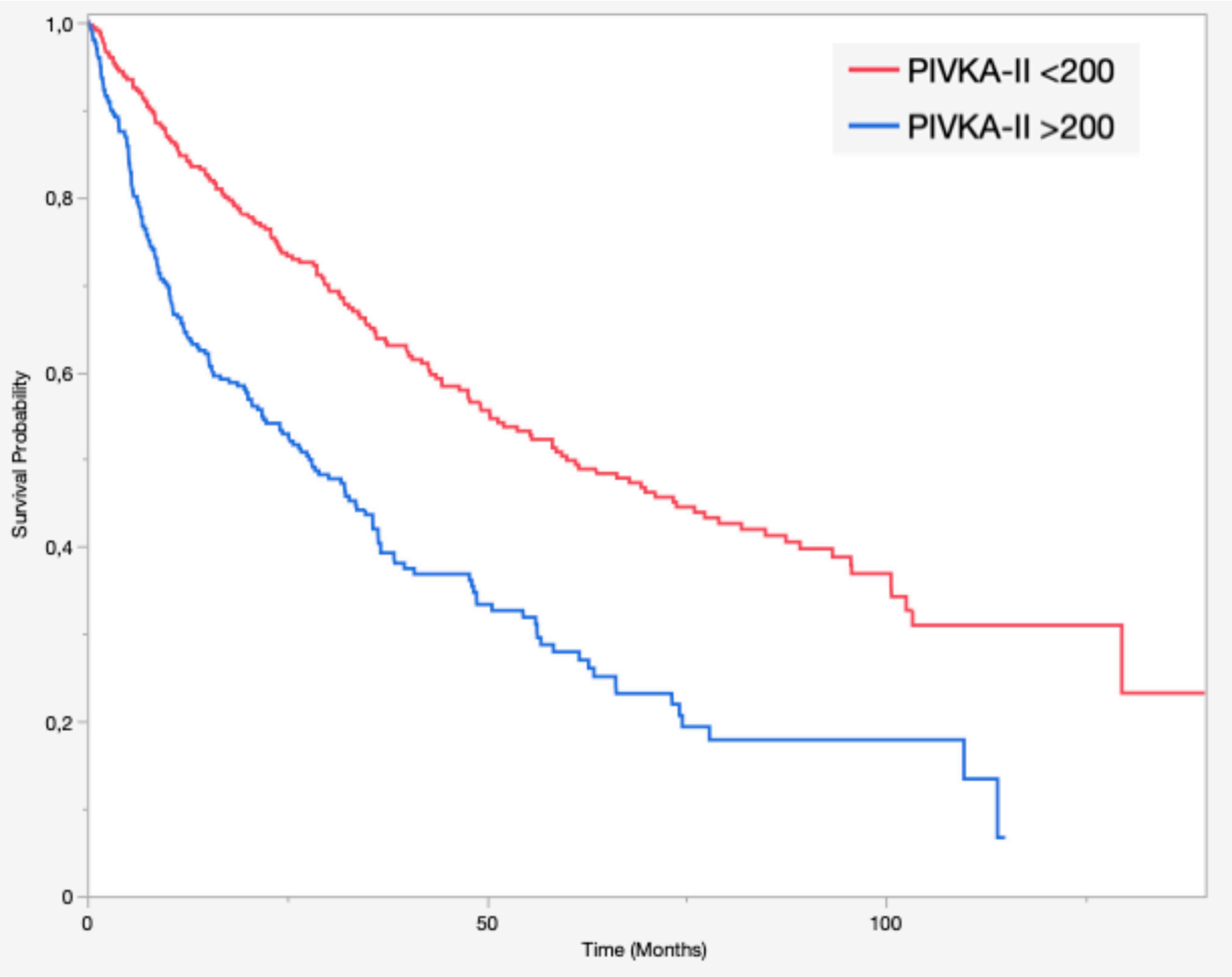

Figure S1b. Kaplan–Meier estimates of overall survival according to baseline PIVKA-II levels. Patients were stratified by PIVKA-II <200 mAU/mL and ≥200 mAU/mL. Time is expressed in months. Survival probabilities were estimated using the Kaplan–Meier method.

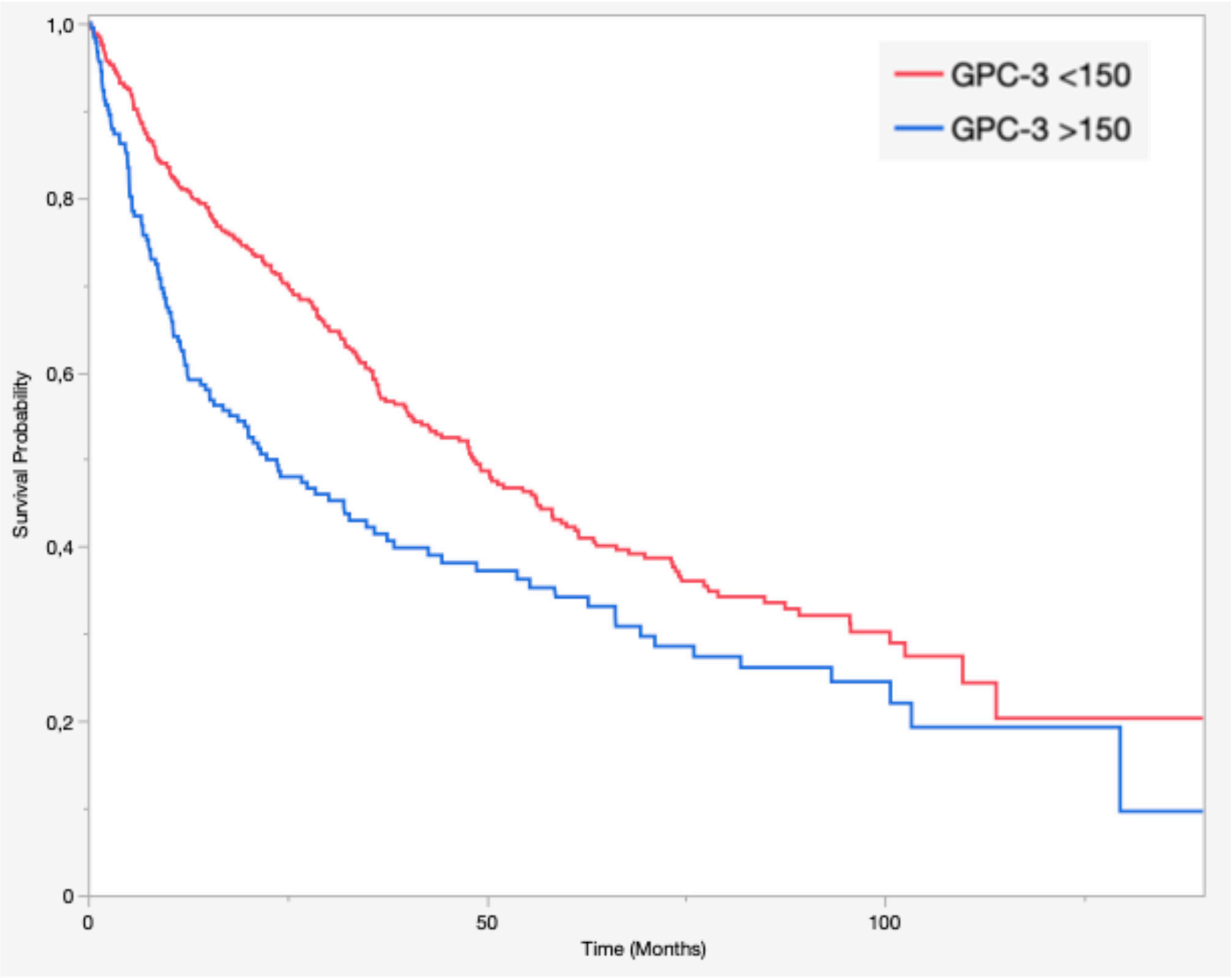

Figure S1c Kaplan–Meier estimates of overall survival according to baseline GPC-3 levels.
Patients were stratified by GPC-3 <150 pg/mL and ≥150 pg/mL. Time is expressed in months. Survival probabilities were estimated using the Kaplan–Meier method.

		Schoenfeld p-value
ALBI grade	1	0.015
	2-3	
BCLC	0	0.260
	A	
	B	
	C	
Ascites	No	<0.001
	Yes	
AFP	<20 ng/ml	0.153
	>20 ng/ml	
PIVKA	<200 mAU/ml	0.509

	>200 mAU/ml	
GPC-3	<150 pg/ml	0.025
	>150 pg/ml	

Supplementary Table S5a. Proportional hazards assumption in the primary multivariable Cox model

Variable		HR	95% CI	p-value
	A vs 0	1.67	1.09-2.54	**0.018**
BCLC	B vs 0	2.56	1.63-4.01	**<0.001**
	C vs 0	4.60	2.79-7.57	**<0.001**
PIVKA-II	> 200 mAU/mL	1.41	1.12-1.79	**0.004**
AFP	>20 ng/mL	1.06	0.83-1.36	0.623

Supplementary Table S5b. Multivariable Cox sensitivity analysis stratified by ALBI grade, ascites, and GPC-3. The sensitivity model was stratified by ALBI grade, ascites, and GPC-3 because these variables showed evidence of non-proportional hazards in the primary model; therefore, HRs were not estimated for the stratification variables. After stratification, no evidence of violation of the proportional hazards assumption was observed for the estimated covariates (global Schoenfeld test p=0.394).
